# ´Feet are second class citizens`: exploring the perceptions of Scottish and Portuguese older adults about feet, falls and exercise- a qualitative study

**DOI:** 10.1186/s13047-020-00434-8

**Published:** 2020-11-11

**Authors:** Monserrat Conde, Gordon J. Hendry, Jim Woodburn, Dawn A. Skelton

**Affiliations:** School of health and Life Sciences, Glasgow Caledonian University, Govan Mbeki Building, G4 0BA, Glasgow, Scotland, UK

**Keywords:** Feet, Falls, Foot care, Exercise, Cross-cultural research

## Abstract

**Introduction:**

Foot problems are likely to contribute to falls risk in older adults. Foot and ankle exercises may be beneficial, but uptake may be influenced by cultural factors. Few studies have explored the views of older adults from different cultural backgrounds about foot-specific falls risk factors, and foot and ankle falls prevention exercises.

**Objectives:**

To explore the views of Scottish and Portuguese community-dwelling older adults who have experienced a fall, about any foot risk factors for falls, and foot and ankle exercises.

**Methods:**

Cross-cultural qualitative study with (*n* = 6) focus groups exploring the perceptions of Scottish (*n* = 10, mean age 76 yrs) and Portuguese older adults (*n* = 14, mean age 66 years) aged, applying thematic analysis.

**Results:**

One main theme **`**evolving awareness about feet and falls prevention´ and three subthemes; (i) Feet are often forgotten, (ii) the important role of footwear, (iii) need to look at my feet and do the exercises were identified. Scottish participants had more experience of falls prevention but there was a lack of knowledge surrounding foot-specific falls risk factors, and the role of ankle and foot exercise in the prevention of falls. Portuguese participants exhibited a fatalistic approach to falls.

**Conclusions:**

Older adults from both nations had little knowledge of foot-specific falls risk factors, being initially unaware of the functional status of their feet and of the role of exercise in foot care and falls management. There were differences between national groups that should be accounted for when developing culturally adequate interventions.

## Background

Ageing brings changes to foot health and foot function [1], with a greater prevalence of foot problems in later life [2]. Hallux valgus, lesser toe deformities, and foot pain become common foot problems and these may increase falls risk [3]. Foot care seeks to address symptoms and prevent foot problems, and includes treatments ranging from nail care to foot and ankle exercises [4]. Furthermore, management of foot complaints and understanding foot related exercise may reduce the risk of falls in this population group [5].

Qualitative evidence shows that self-footcare is perceived as challenging for both healthy [4] and condition-specific groups of community-dwelling older adults [6]. Knowledge and awareness on general foot health and foot function also appears to vary greatly in this age group [4, 6]. Nonetheless, there appears to be a gap in the literature concerning specific perceptions surrounding the link between foot problems and falls risk.

Little is known about older people’s decision-making process regarding exercise for falls prevention, particularly from culturally and linguistically diverse backgrounds [7, 8]. Although falls prevention seems to be a low priority for older people in the United Kingdom, as they rarely perceive falls can be prevented with exercise, there are differences in beliefs and attitudes between ethnic groups [8, 9]. Jang et al. (2016) found that cultural values and beliefs had a profound and lasting impact on how older adults perceived and defined ageing and health, their outlooks of cultural appropriateness, family obligations and gender roles and the connotations they attributed to exercise and fall prevention strategies [7].

Falls prevention interventions need to be culturally suitable, as programme participation is influenced by cultural and motivational, social and environmental values [10]. Negative cultural perceptions result in a wide range of implications from hiding falls episodes, to rejecting walking aids or deeming exercise as not age or gender appropriate [7, 10]. The meaning of exercise also displays a great cultural variability, having been shown to influence an older people’s engagement in exercise-based falls prevention interventions [7]. Therefore, it’s important to consider those factors to confirm evidence-based recommendations and interventions [10].

To the best of our knowledge, there is a lack of comparative research concerning older people’s perceptions about falls prevention across European countries [11]. It is well known that the effectiveness of falls prevention interventions depends on the uptake and adherence rates [12] . Given that these appear to be influenced by the beliefs, perceptions and preferences of older adults [7, 13, 14], it seems relevant to ascertain any underlying cultural variation within the European context. Particularly, considering that variations of ageing perceptions, within social contexts, have been already identified on the north-south European axis [15].

Furthermore, the available qualitative evidence does not address the views of older adults surrounding foot related falls risk factors and foot care and the role of falls prevention strategies such as exercise. The aim of this study is to explore the views of Portuguese and Scottish community-dwelling older adults, who have experienced at least one fall in the past year, about these topics.

## Methods

### Study design

This qualitative study using focus group discussions was part of a larger programme of research exploring older adults´ views on a tailored home-based lower limb, ankle and feet exercise programme [16]. After participants took part in an introductory group session and a one-week trial of the exercise programme; three focus groups were conducted in Scotland and three in Portugal. Further information about the home-based exercise programme, views of participants on undertaking the programme and the outcome measures proposed are reported elsewhere [16]. Focus groups were used as this approach offers high ecological validity, and is adequate for groups less experienced in research participation [17]. All advantageous features when conducting cross-national research.

### Participants and recruitment

Scottish and Portuguese community-dwelling older adults, aged ≥60 years, who had at least one fall in the previous 12 months were invited to participate. Participants were recruited via a variety of convenience sampling methods through local community-based organisations for older adults [gatekeepers- (e.g. ROAR connections for life-UK, Envelheseres network -PT), snowballing] and also through a newspaper advertisement (UK). Recruitment strategies differed between nations due to contextual factors (e.g. country-specific differences in how gatekeepers manage access to prospective participants) and available resources.

### Eligibility criteria

Participants were required to be able to ambulate and travel independently to venues, able to read and write in English (Scotland) or in Portuguese (Portugal). Participants were excluded if they were diagnosed with central nervous diseases, cognitive impairment, self-reported depression, significant visual impairment or registered blindness, recent lower limb fractures (< 6 months), recent joint replacements (< 1 year), or other medical contraindications that would not allow participants to exercise safely (e.g. acute medical condition)**.**

### Data collection

A semi-structured interview guide was used [see Additional file 1 Appendix 2, Supplementary Data]. Its structure was inspired by interview guides used in previous research projects [18, 19]. Focus group interviews were adaptable to the participants‘ contributions, allowing further questioning [20, 21]. One researcher (MC), trained in this approach, led the interviews with support for flipchart note taking by another researcher (UK) or assistant (Portugal-PT). For data validation, participants were asked if notes in flipcharts expressed their views accurately. Focus groups took place in a meeting room either at the University (UK) or at the community centre (PT) and lasted between 45 and 120 min. Focus group discussions were digitally recorded using a sound recorder (Model MICD-UX560 Sony™).

### Qualitative data analysis

All focus group audio recordings were transcribed verbatim and anonymised [20, 21]. The anonymised Portuguese transcripts were translated to English, and then proofread by a certified translator. Data was uploaded and managed in NVIVO 11™ software. Qualitative analysis followed the six phases of thematic analysis framework defined by Braun & Clarke [20]: 1) Familiarization with the data, 2) Generating codes, 3) Generating themes, 4) Reviewing potential themes, and 5) Defining and naming themes. This qualitative analysis approach was selected as it captures people’s unique views on the researched topics and is also suitable to analyse data derived from focus groups [20]. Flipchart and field notes were considered alongside the transcripts. Three researchers independently analysed 3 randomly selected transcripts each in order to enhance validity of the researcher’s coding procedures through collaborative discussion. Minor discrepancies in terms of the procedures were resolved between the team. The researcher then developed the coding frame for all transcripts, which informed her search for initial themes. These were reviewed in agreement with the research team. Illustrative quotes of the theme and subthemes were extracted from the data.

## Results

### Participants

15 Scottish and 24 Portuguese participants agreed to enrol in the study, but focus group analysis only included 10 and 14 participants respectively, due to a variety of reasons [see Additional file 1 appendix 1, supplementary data].

Ten Scottish adults (mean age 76 years; range 68–80 years, 7 women), and fourteen Portuguese adults (mean 66 years; range 64–74 years, 12 women) participated in this study. The three focus groups in Scotland took place, with 3, 3, and 4 participants respectively. In Portugal, the focus groups occurred in three different small size cities, with 2, 6 and 6 participants each.

Socio-demographic characteristics of the participants are presented (Table 1).

**Table 1:** Characteristics of participants

Characteristics		Portugal	Scotland
Age	Median (P25; P75)	66 (64; 74)	76 (68; 80)
Sex			
Male	*n (%)*	2 (14.3)	3 (30.0)
Female	*n (%)*	12 (85.7)	7 (70.0)
Occupation			
Retired	*n*	9	10
Worker	*n*	5	0
Education			
Primary school	*n*	6	0
Secondary school	*n*	8	4
College diploma	*n*	0	4
University degree	*n*	0	2
Number of falls in last 12 months	Median (P25; P75)	2 (1; 3)	2 (1; 4)

**Fig. 1:**
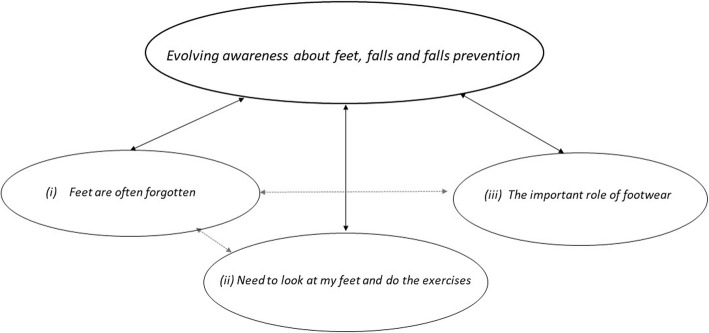
Theme and subthemes

Qualitative findings were categorized into one main overarching theme and three subthemes (Fig. 1). Supporting quotes for each theme are presented as (pseudonym, Focus Group number and Country (UK/PT)). Further illustrative quotes for each subtheme can be found in Appendix 3, Supplementary Data [see Additional file 1].

### Evolving awareness about feet, falls and falls prevention

This theme encompasses the views expressed by focus group participants regarding foot health, foot function and falls, both independently and in relation to each other; as well as to their general health and functional status. Both national groups appear to lack awareness and previous knowledge about these topics, particularly the Portuguese participants.

There was a lack of awareness surrounding foot health, foot function, foot related falls risk factors and foot care management. Participants admitted that feet were largely neglected.

*“They pamper their hands, they pamper their faces, they pamper everything else (…) But mostly the Feet… they are second class citizens.”* (Anthony, FG3_UK)

It was evident through discussions with participants that there was limited understanding between the link between falls risk and foot related issues. This was highlighted while participants were recalling their individual falls events, they had not considered that their feet could have been a contributing factor to their fall.

*“Well…I tell you what I thought: Nothing! Not at all about my feet. I’ve thought it was something either in my head or my ear…”* (Lisa, FG2_UK)

Poor awareness on these topics was potentially derived from lack of information. Even the Scottish participants who had been enrolled in falls prevention programmes did not recall being given information on foot-specific falls risk factors.

*“The feet are there and we use them and we don’t think about them. Therefore this has been quite an eye opener.”* (Louise, FG1_UK)

Participants did sometimes elaborate on their reflections about specific foot falls risk factors.

*“I think that if the foot is a healthy foot that has support...and even...I mean...even if one loses balance...But can regain balance better than if...Even if the legs are good, but the foot isn’t... One falls more easily. I think!”* (Julia, FG3_PT)

A fatalistic perception of falls was conveyed by quite a few Portuguese participants, attributing these events to fate or chance. The belief in a protective external force was also portrayed.

*“No! Thank God! I didn’t fall this week! Oh my God! Thank God!”* (Mariana, FG1_PT)

Such comments were sometimes accompanied by hands in praying position, or followed by the cross sign.

It was also common for Portuguese participants to depict their falls as unexplainable, as they appeared to not be aware of any main causes that could be triggering such events. This seemed to be closely related to lack of information on the topic, which could be derived from either not recalling or not being given any explanation by health professionals.

*“I fall often, but (...) I don’t know…I don’t really know how to explain it… I don’t need to stumble on anything. Nothing. And when I feel it (….) It’s happens whenever.”* (Vera, FG3_PT)

*“I don’t know…No one has ever told me, and I don’t have knowledge for those things…But I question myself…”* (Sofia, FG3_PT)

#### (i) Feet are often forgotten

Neglecting their feet appeared to be rooted in a combination of aesthetic, social and functional expectations. Foot care was described as reliant on other people’s aesthetic judgement of body appearance. Since feet are often covered by footwear, and not on display, they ended up being less prioritized.

*“(…) your feet are poor relations in your body, you know? no one sees your feet”.* Anthony (FG3, UK)

However, those who perceived their feet as key elements to perform valued activities of daily living (e.g. sports) acknowledged their importance. Interestingly, these participants would portray themselves to be an exception to the norm.

*“I’ve always been aware of foot health because I look on it ‘If you’re not on your feet, you’re in your bed!’”* (Lisa, FG2_UK)

Across both nations, participants generally seemed unaware of how relevant feet are to human movement unless they had foot problems (pain or deformities). Most undertones around feet tended to be negative, with participants picturing themselves as having ´bad feet`.

*“(..) all deformed…and the toes are like this, they don’t lift up”* (Sofia, FG3_PT)

Feet were mostly either ignored or perceived as a source of discomfort, in both national groups. Foot problems appeared sometimes to be the main motivators for individuals to take any measures regarding foot health and foot function.

*“Yes. because they’re there and mine haven’t broken...So I hadn’t anything to think about it”* (Louise, FG1_UK)

*“No! I didn’t think that because I don’t have other than cramps I don’t have a lot of stuff in my feet”* (Ana, FG1_PT)

Regarding participants´ perceptions about foot care, these mainly concerned hygiene with some mention of skin and nail care.

*“(…) They’re there…They get washed and they cleaned and that’s it. ((Laughter))”* (Louise, FG1_UK)

Care towards feet was more apparent in those with pre-existing problems (e.g. diabetes mellitus). These participants also demonstrated relatively more knowledge of other aspects of foot health (e.g. sensitivity), due to regular health check-ups.

“*I have been advised about foot health because I am a diabetic.”* (Catarina, FG3_PT)

The Portuguese focus groups displayed overall more negative perceptions, with negative internal representation of feet. Sometimes fear of movement and low self-efficacy derived from previous injuries would come across participants´ remarks:

*“(..) And when it hurts, I say alright! I am not going to walk more. I’m going to stop it here. The pain is on the foot, but the head thinks.”* (Vera, FG3_PT)

Interestingly, there was an initial lack of awareness with participants from both nations on the range and type of movement available within the foot. The limited understanding regarding the functional abilities of the foot as well as recognising the complexity of movement within the foot was evident across cultures.

*“I think when you know how you have to exercise your feet you (are) suddenly aware of how similar they are to your hands…Same bones (…) And I am horrified of how poor my feet are.”* (Louise, FG2_UK)

*“Between tip toes and heel I didn’t…I mean, I didn’t think that it was as important as it really is.”* (Catarina, FG3_PT)

In fact, going through the experience of doing the exercises and discussing them was portrayed as enlightening in many ways:

*“You know? (…) you take your feet for granted that you never give them a second thought…But this made you think about it”* (Sarah, FG3_UK)

*“No, I didn’t think about it a lot. No one had explained it to me before. That walking on your tip toes or heels could be so important for balance*.” (Leonor, FG3_PT)

This subtheme has emphasized how older adults hold a wide range of views about constructs surrounding feet, including self-awareness regarding movement and function, discomfort, and aesthetic and social features. Negative views were prevalent across national groups, particularly in Portugal.

#### (ii) Need to look at my feet and do the exercises

Participating in the study appeared to influence older people’s views on the value of specific ankle and foot exercise and self-management. Many admitted it had changed their awareness of the functional status of their ankle and foot complex.

*“It has raised awareness of the need to look at my feet and do the exercises. (...) I hadn't really thought very much about what we needed to do... And this was a wake-up thing”.* (Louise, FG1_UK)

Most focus groups´ members had not been aware that such exercises could be integrated into a falls prevention plan:

*“I also didn’t, right, didn’t know that these exercises were beneficial for falls and that.”* (Ana, FG2_PT)

Overall, participants were not used to doing specific foot exercises and expressed frustration at times with how challenging moving their toes could be.

*“My toes are all crippled (...) they don't lift up. Lowering even less so”* (Sofia, FG3_PT)

Foot problems (e.g. toes deformities, arthritis) also posed an additional challenge to some of them.

“*And I’ve got arthritis…Well it’s not severe…but doing the exercises was actually heavy going for me…on the barefoot ones…*” (John, FG2_UK)

After they had tried the exercises, the idea of lower limb, ankle and foot exercises being beneficial to foot movement and feeling more stable, was repeatedly expressed by participants of both countries.

Some participants mentioned immediate improvements from the one-week trial, which seemed to relate with a sense of increased self-efficacy. Many participants appeared more confident of being able to self-manage their functional status, which helped with overcoming any initial difficulties that they had.

*“It’s just the fact that my toes didn’t do it, sit down and think about it, lack of use…Therefore get on and do something about it.”* (Louise, FG1_UK)

The idea of ´having a problem and doing something about it´ appeared to resonate across focus groups.

Other participants reported improvement of confidence, function or symptoms, such as pain, following doing the specific ankle and foot exercises.

*“I mean… even in a week (…) I feel as if I’m stronger.”* (Lisa, FG2_UK)

*“The foot arch…These [the exercises] helped me…Helped with the pain that I had to go away…”* (Catarina, FG3_PT)

Many participants demonstrated recognizing improved motor skills, but also being aware that their maintenance and improvement needs consistency over time.

*“Right, but this we need to continue doing it afterwards. I couldn’t lift my big toe either. And now I can…I can.”* (Júlia, FG2_PT)

Links between disuse, functional importance of strength training and specificity of certain movement patterns could be traced across focus groups. Balance training was associated with an ability to better perform activities of daily living.

*“So, if someone does these exercises, improves their balance, mobility to…To being able to walk better and have a different balance. That’s what I think”* (Vera, FG3_PT)

At times, participants clearly expressed believing that these exercises could contribute to maintaining their functional status the longer term, including their ability to walk independently:

*“The stronger your legs get, the less chance you’ll need a Zimmer frame!”* (Thomas, FG2_UK)

Even the most active participants seemed to identify specific functional gains that could be obtained from the exercises. They recognised that these would be working different muscles groups and attributes, completing other physical activities. Interestingly, these more in-depth reflections were more evident in the Scottish than Portuguese focus groups.

*“Although I think I’m like you, I’m quite active and I am out everyday walking. You’re not using the same muscles…You don’t feel you’re using the same muscles as the exercises pinpoint…They pinpoint the muscles being used”* (Jean, FG2_UK)

This subtheme renders a growing understanding of the relevance of specific exercises as part of foot care by the participants, awareness of their own foot function; self-perceived value and implications of specific exercises within a falls prevention scope. These were common underlying experiences in both national groups, despite an apparent lower physical literacy by Portuguese participants.

#### (iii) The important role of footwear

Participants spontaneously brought up the role of footwear in their discussions. Footwear preferences were mainly driven by a search for comfort, without any specific guidance from health professionals. Even in the presence of health conditions such as injury or joint deformity.

*“No I’m the one who has to check what fits me”* (Leonor, FG3_ PT)

Several participants also expressed adjusting their footwear choice over the life course, as result of the ageing process.

*“It’s been many years since I’ve only used flat shoes”* (Leonor, FG3_PT)

Scottish participants discussed more the idea of what “safe” footwear was in relation to their capabilities.

*“Aye. Have any of yous ever fell when ye had high heels on? Shoes with high heels?”* (Thomas, FG2_UK)

*“I don’t wear high heels now. I wouldn’t trust myself to be honest with high heels…Because I go over my ankle quite a lot… (…)”* (Jean, FG2_UK)

Despite exhibiting some concerns around footwear safety, inquiring about wearing loose slippers and being barefoot, participants were slightly surprised that footwear could influence falls.

*“I wasn’t aware of the role…The important role of footwear.”* (Elaine, FG1_UK)

An increased awareness that footwear could influence normal foot function, potentially increasing falls risk, appeared to arise from participating in this study for both national groups.

*“you brought up a point that I find with these… super comfortable [brand of trainers with gel insoles] …But I… If they stick, I do tend to go forward. I’m not lifting my foot, obviously, high enough. Because it’s only since I got these that I’ve noticed that”* (Jean, FG2_UK)

Participants offered mixed remarks about being barefoot. Portuguese participants admitted to being barefoot more often and were more likely to wear sandals or flip flops because of the weather. Interestingly, different to the Scottish focus groups, no considerations about footwear preferences and falls episodes were expressed by them.

This subtheme shows how participants perceived footwear as a coadjutant to maintain/improve foot function, its contribution to falls risk; and both their reasoning and preferences when choosing shoes in their daily lives. Contextual differences between nations played a role on the latter.

## Discussion

This is first qualitative study to explore community-dwelling Scottish and Portuguese older adults´ views about feet within a falls prevention scope. It is also the first study to offer insight into Portuguese older adults´ perceptions about falls and exercise-based falls prevention. The main focus of the findings were participants´ perception of their feet and how this links to understanding risk factors associated with falling and managing/minimising this risk potentially through specific foot exercises. It provides key information concerning a lack of awareness towards foot related falls risk factors and self-foot care within a falls prevention scope; including specific foot and ankle exercise in both national groups. Portuguese participants expressed more fatalistic views about falls, with some typifying their falls as unexplainable. This national group was also apparently less aware and informed that exercise could prevent falls.

A lack of awareness of falls related risk factors associated with their feet was predominant across both nations, even among Scottish participants who had previous experience with exercise-based falls prevention programmes. A recent meta-analysis suggests that foot problems are associated with falls in older adults; recommending health screening and specific referral within falls assessment [3]. Most participants mentioned having foot problems, which meant that they could potentially benefit from this information to their falls risk.

Portuguese participants presented more fatalistic views about falls; concurring with previous literature about other national groups [8, 9, 22]. *Fado* (destiny or faith) and catholic doctrine are rooted in Portuguese culture. Although religious beliefs can contribute to developing a feeling of acceptance of falls, they can also act as a coping mechanism for older adults [8]. Faith-based social networks can also facilitate exercise engagement [23]. These could be considered when planning programmes in Portugal.

Some Portuguese participants characterised their falls as *unexplainable*, similar to older adults with visual impairment [24]. They were at a loss as to why they would fall, emphasizing that they had not received any information from health professionals. There are several reasons why participants may perceive that falls are not preventable or that they have not been provided with falls prevention education. Previous research has shown that older adults may not perceive themselves to be at risk of falling [9, 25, 26], believe that falls are not a medical issue [27] or just assume that they are an inevitable consequence of ageing [25]. Believing that they should be more careful to avoid falls [8], dismissing non injurious falls [27] or not wanting to burden family members [7, 22] also appear to influence reporting of falls. Discussing falls may also cause anxiety [27], being perceived as signs of functional decline and frailty [25]. All these aspects can contribute to older adults not discussing the issue of falls with health professionals [9, 25–27].

Concomitantly, health professionals also face several barriers to provide guidance to older patients regarding falls prevention and management, ranging from patient influence, to education training, to communication between staff and patients [28]. The lack of structured fall prevention guidelines and insufficient training has also been identified as a major barrier to health professionals providing advice on exercise to prevent falls [29], which may be relevant in countries such as Portugal where there is not an established nationwide NHS guidance. Additionally, older adults often present competing clinical priorities with urgent medical issues being prioritized over falls in the short time that health professionals have available to provide care [30]. Finally, it should also be noted that health messages may sometimes be misunderstood, or forgotten, by patients [31, 32]. This might have also been reflected in our findings.

Interactions with health professionals can positively or negatively influence the uptake of falls prevention interventions, with older adults being more likely to participate in an exercise-based intervention if they are referred or encouraged to participate by a health professional [27]. Our findings appear to align with these findings, also highlighting that there is an opportunity for clinicians to address foot-related falls risk factors with their older patients. These aspects should be further researched from both the public and health providers´ perspective and contemplated when planning interventions.

Participants admitted that feet are less prioritised than other body parts, agreeing with previous research [4]. Feet not being on display was the main reason given for this ´neglect´. In our study, feelings of privacy were not mentioned by participants, unlike Miikkola et al’s findings [4]. Appearance and body image are important to older adults, as they signpost personal identity, physical functionality, and social status; they are also influenced by sociocultural stereotypes of the ageing body [33]. Older adults may become self-conscious of age-related changes to feet morphology and appearance [1], leading into overlooking them further. In fact, negative views of feet were common across focus groups, particularly among Portuguese. Foot problems were managed rather than prevented, a behaviour previously highlighted among other condition-specific and national groups of older adults [4, 6, 34]. Foot pain, discomfort and other problems were common complaints, concurring with strong quantitative evidence [35]. These were also the main motivators to seeking a medical appointment for foot health [4, 34, 36]. Previous research has identified the importance of positive perceptions of healthcare to facilitate older adults´ future foot consultations [34, 36]. Our study reflects the same trend in both nations emphasizing the need for education.

Sometimes participants with a history of foot health problems admitted being protective of their feet, which would trigger some fear and avoidance of movement [37]. These potential manifestations of lower self-efficacy can perhaps relate to the fear-avoidance model of pain [38]. Reinforcing the role of health professionals in educating older adults about foot health and foot function management [4, 6, 34], encouraging regular movement [39] and patient-provider communication processes [40, 41] are important mediating agents in pain-related outcomes. Our findings also suggest that foot related issues could perhaps be further explored within the patient-provider communication processes.

As with Miikkola et al. [4], our participants lacked knowledge of proper foot care which can negatively influence their foot health and foot function, and consequently their foot-specific falls risk factors. Only participants who had foot problems, or who were at a higher risk (diabetes mellitus) reported having received health information about foot self-care through their healthcare services [6, 34]. However, assumptions about the quality of self-foot care cannot be made, as this was not explored in depth. This study found that specific foot and ankle exercises were not considered as a strategy for foot health, contrasting with other studies [4]. Most Scottish and Portuguese participants were unaware of the role of these exercises in falls prevention [5], which had not been previously reported. Moreover, Portuguese participants had very little knowledge that exercise could prevent falls. Similar conclusions were found regarding other national and ethnic groups [7, 8, 10], but it is new for Portuguese older adults. Differences in the baseline characteristics of participants and healthcare resources between nations may have influenced our findings. Low health literacy and low education levels are predominant among Portuguese older adults [42], which may have also contributed to the findings of this study. Our findings also suggest possible lower physical literacy among Portuguese participants [43].

Some participants mentioned immediate benefits during the one-week trial (e.g. pain reduction, improvement of muscle strength, increased confidence). This may be a result of placebo effect related to exercise, particularly for those with negative beliefs about their physical abilities [44]. Furthermore, such self-perceived improvements may be coincidental. Motor learning may also have contributed to self-efficacy [45], as participants reported observing an improvement in their motor performance. An increased self-awareness of foot function was often mentioned, with many reporting an improvement in observed motor function, potentially demonstrating responsiveness to the neuromuscular effects of specific ankle and foot exercises. This was shared across national groups, independent of previous experience with physical activity or exercise-based interventions, and it has not been previously reported in the literature; possibly, this may have been a manifestation of increased physical literacy [43].

Focus groups reported that a specific exercise programme could improve the available range of motion, muscle strength, and balance in the longer-term, linking these as key abilities to performing their activities of daily living. The latter was highly valued, conforming with identified enablers of engagement in exercise-based falls interventions [46]. Scottish participants appeared to be more aware of the specificity of these ankle and foot exercises, recognising extra functional elements to their regular physical activity routine. Portuguese were less expressive of specific contributions. Differences in physical literacy may explain these differences; the Scottish participants were overall more educated, more physically active, and considerably more experienced with exercise, all characteristics that contribute to greater levels of physical literacy [43]. However, Scottish participants also appeared to have increased their physical literacy in relation to foot function as a result of this study.

Preferences of footwear varied between nations due to weather conditions. Overall, participants expressed being originally unaware that footwear could influence falls, albeit being very curious about optimal footwear for foot health. Participants expressed that shoes should be individualised to their needs and comfort, aligning with existing evidence [4]. Most reported that they had not received any guidance about optimal footwear, with all admitting never being informed about footwear as a risk factor for falling. Footwear has been considered to possibly impact fall risk [47], however, there is no conclusive evidence supporting a causal relationship between specific types of footwear and falls rates in healthy older adults [48]. Nonetheless, footwear impacts older adults´ foot health [49, 50], including increasing the chances of acquiring foot problems (e.g. hallux valgus) associated with an increased falls risk [3].

### Implications for intervention development

Our findings support the emphasis on regular educational components surrounding foot-related falls risk factors as part of specific ankle and foot exercise interventions for older adults. Participants exhibited a generally low physical literacy related to foot function and specific ankle and foot exercises, these should be assessed and targeted by robust evidence-based educational components.

Falls prevention interventions developed for Portuguese older adults would benefit from accommodating the needs of a population with lower health and physical literacies. This may translate into reinforcing general falls educational components as well as providing greater individualized support when introducing exercise-based interventions to older adults.

## Limitations and future research

This study has some limitations. Recruitment varied between nations due to contextual factors. Selection bias may have resulted in recruiting individuals more interested in these topics. It was not possible to interview those who declined to participate or dropped-out. Differences in characteristics of national groups (particularly age, education and current employment), and contextual settings, could have influenced our findings. Nonetheless, we used adequate approaches for each national setting and were able to capture cultural undertones across the data.

Participants had tried a lower limb, foot and ankle exercise programme for a week. Exploring views separately from participation in a programme may better differentiate between baseline and acquired knowledge, in future studies.

Future research should also explore the views of health professionals, involved in falls management, about the role of foot related conditions and their relationship to falls risk, as well as exercise-based interventions to assist reduce the risk of falls in older people. Resulting evidence could inform clinical practice. More qualitative studies exploring Portuguese older adults´ views about falls prevention would enable culturally adequate evidence to inform national public health policies.

## Conclusions

By performing a thematic analysis of focus group interviews, we found that Scottish and Portuguese older adults had little knowledge of foot related risk factors associated with feet, being unaware of the functional status of their feet and of the role of specific lower limb, ankle and feet exercise in foot care and falls management. Feet were described as neglected body parts, with problems being managed rather than prevented. Footwear was not considered for reducing falls risk. Participating in the study contributed to an evolved awareness about these topics. Furthermore, Portuguese participants demonstrated little knowledge about general falls-risk factors and falls prevention strategies, with some displaying a fatalistic outlook, or considering falls to be unexplainable. Such aspects could be considered when developing culturally appropriate interventions.

## Supplementary Information

**Additional file 1.** Appendix 1 includes the recruitment flowcharts for both nations, appendix 2 is the focus group interview guide, appendix 3 includes more illustrative quotes for the theme and each subtheme, and appendix 4 is a completed reporting checklist for qualitative studies.

## Data Availability

The data excerpts supporting the conclusions of this article are included within the article (and its additional file 1). Further anonymized data may be available on reasonable request from the corresponding author. The original focus groups audio recordings are not available to ensure the anonymity and confidentiality of the participants.

## Abbreviations

UK: United Kingdom
PT: Portugal
FG: Focus Group
NHS: National Health Service
