# Supplementary material for: ´Feet are second class citizens`: exploring the perceptions of Scottish and Portuguese older adults about feet, falls and exercise- a qualitative study
Source: J Foot Ankle Res. 2020 Nov 11;13:66. doi: 10.1186/s13047-020-00434-8 (PMC7659063; doi:10.1186/s13047-020-00434-8)
Supplement: Supplementary file 1 — Additional file 1. Appendix 1 includes the recruitment flowcharts for both nations, appendix 2 is the focus group interview guide, appendix 3 includes more illustrative quotes for the theme and each subtheme, and appendix 4 is a completed reporting checklist for qualitative studies. [file 13047_2020_434_MOESM1_ESM.docx]

**Appendix 1 – Recruitment flowcharts**


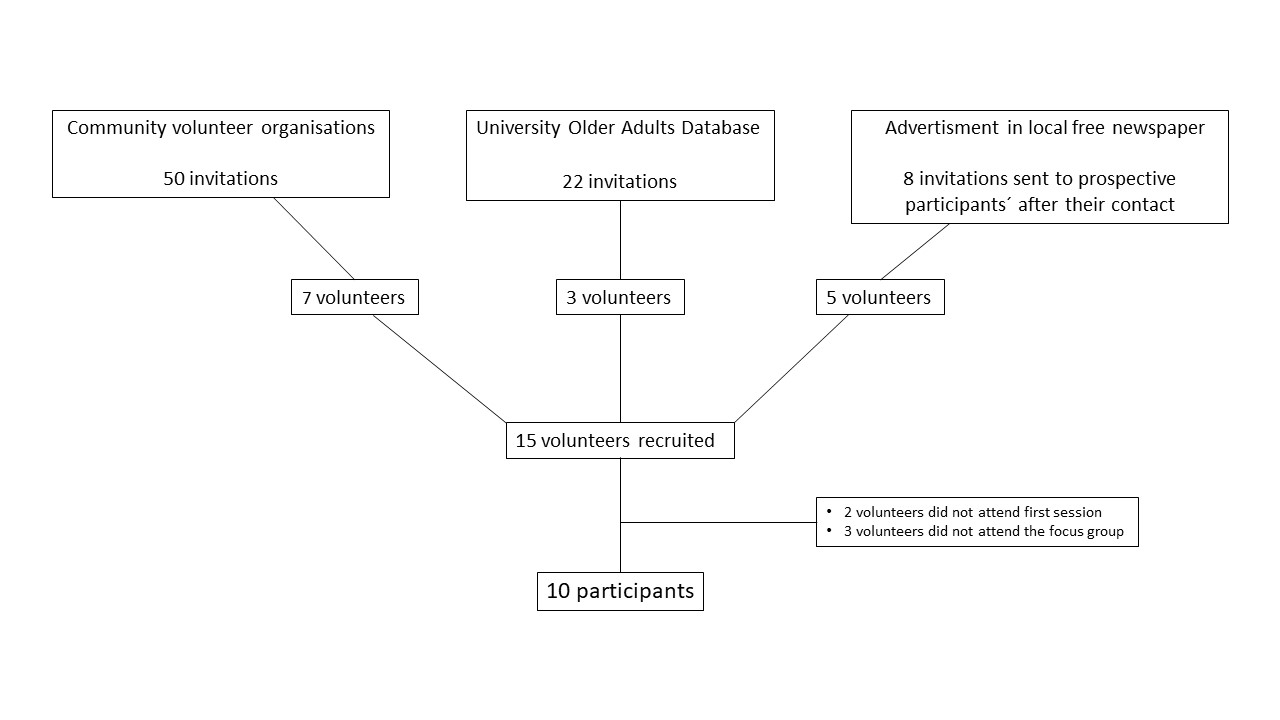


Figure 1.1 Recruitment in Scotland


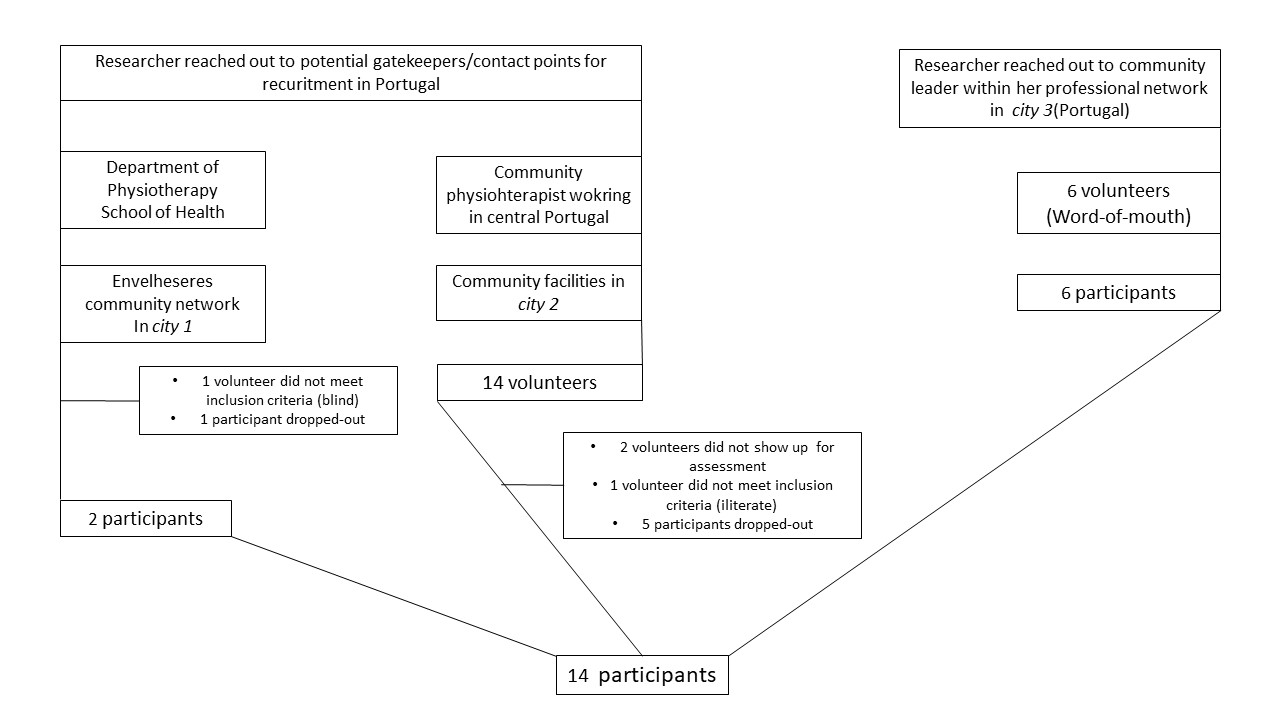


Figure 1.2 Recruitment in Portugal

**Appendix 2- Focus group interview guide**

(All identifying information was removed from the introductory text)

**Introduction to the focus group and warm-up**

**Topic 1: Assessment session**

**1.1 *All of you underwent an assessment session where you answered four questionnaires and did four functional tests. Do you remember? (Briefly describe the instruments and tests if needed) I am going to ask you a few questions about them.***

- How did it feel to undergo the assessments? (Prompt if no response -? tiring, learned something new, easy, hard? don’t know what they were for? Embarrassing? Uncomfortable?)

- What did you think about the questionnaires? (Remind them of which ones) Did they seem relevant to you? Did you agree/disagree with any of the questions?

- What about doing the functional tests? (Remind them of which ones) How did you feel when being tested?

**Topic 2: Home-based lower limb and ankle exercise programme**

***2.1 You were first introduced to this exercise programme by means of a supervised exercise group session. I would like to ask you a few questions about this first session I did with you, showing you the exercises.***

- What were your perceptions of the role of your feet in relation to unsteadiness when standing or walking before the exercise programme?

- Did this session change your perceptions or views about how you’re your feet relate to feeling unsteady when doing your activities of daily living?

- If yes, how?

- Was there anything you learned about the benefits of foot exercises to prevent falls that you were not aware of before doing this session?

- If yes, what?

- What are your views about the way the exercises were taught? (Explore any positive or negative views)

- Do you feel confident that you could perform the exercises by yourself after this session? Why (not)?

***2.2 After this group session, you perform the exercises at home during a week.***

**-** Was it easy to remember to do the exercises? Did you do them 3 x per week? (Explore by asking which strategies could be used as reminders).

- Did you find the exercises easy to fit into your daily routine? (Explore barriers/ facilitators)

- What do you think is the best time of the day to perform the exercises? When did you tend to do your exercises? Why?

- What are your views about doing the exercises three times a week? (Explore barriers/ facilitators)

***2.3 Your programme kit included an exercise booklet. The next few questions will explore your views of this booklet.***

- Was the information useful? Do you feel that you learned something by reading it? Why (not)?

- Did the booklet help you when you were doing the exercises at home? (Explore reasons)

- Is the information included in the booklet easy to understand? Would you suggest any changes in terms of language? Was it easy to read? (Explore font, colour etc)

- What are your views on the images that were provided to assist you complete the exercises correctly”? (If there is no answer, explore it further. “Were they helpful?)

- Would you suggest any changes in terms of design to make it more friendly or easy to use?

***2.4 Let’s talk about the physical exercise diary now.***

- Was the diary easy to fill in? Why (not)? (Explore any negative or positive feedback)

- What would make it easier to complete?

- Would you suggest any changes in terms of making it more user-friendly?

***2.5 You received a mid-week phone call where I checked on how you were doing with the programme.***

- What were your views on how useful the phone call was? (Explore any positive or negative answers)

- Did it motivate you to keep doing the programme? Explore any positive or negative answers)

- What were your views about that feedback I gave you on the phone? (Explore any positive or negative answers)

- If the programme went on longer – say 3 or 6 months, how often would you want a phone call? Are there other ways you would want a prompt or reminder? Say by email or text or letter?

**2.6 The actual exercises in the programme**

- Let’s have a look at each one of the exercises. [The moderator will present one exercise at a time and will explore the following points for each one.]

- What do you think about the name of the exercise? Do you have any suggestions for a different name?

- Did you like or dislike this exercise? Why (not)?

- Can you describe to me how easy or difficult you found it to do this exercise”?

- Was it easy to remember how to do this exercise? Why (not)?

- Which muscles or part of your lower limb do you think that you are working when you perform this exercise? (Point to them)

- Do you think that this exercise is useful? Why (not)?

- Would you suggest any changes to this exercise?

**2.7 *We will now discuss some points about the overall programme.***

- Was there anything you particularly liked or disliked about this programme? Why (not)?

- Would you prefer the supervised session at the start to be one to one or was it ok in a small group?

- What are your views about the number of exercises?

- Do you think that this programme could be good for your health? Why (not)?

- How often and for how long do you think you would be prepared to continue to do these exercises yourself? What might help you to continue to do them more often and for a longer period of time?

- Would you be willing to maintain this home-based programme on the longer term (3, 6 or 12 months)? Why (not)?

- Was there anything you particularly liked or disliked about this programme?

- Would you recommend this programme to someone else, for example, your friends or family? Why (not)?

- Do you have any other suggestions on how to improve the exercise programme and how it is delivered?

[Prompt if not mentioned in the earlier section: Did any exercises cause any discomfort? If so which ones? Why do you think these caused you discomfort?]

**Topic 3: Study procedures**

Finally, I have a few quick questions about the study procedures, the information given, paperwork completed and the consent process. We will be undertaking a larger study on this programme in the future, so we want to ensure the study procedures are as easy to follow as possible.

- How clear was the information provided in the Participant Information Sheet? (Was there enough information provided?)

-How easy was the consent form to understand?

- Did you have any problems completing the consent form?

We will want to recruit large numbers of people for the next study.

- Do you have any suggestions for strategies to recruit participants?

**Cooling Down**

- Would you like to add anything else that we have not covered and that you consider relevant to mention? Are there any final comments?

**Concluding Remarks**

Our focus group is coming to a close. Thank you very much for your help and time. You will receive a summary of the main results of this study, by post, once it is completed.

I will now switch off the digital voice recorder.

**Appendix 3 – Illustrative quotes**

| **Theme** | **Sub-themes** | **lllustrative quotes** |
| --- | --- | --- |
|  | **Feet are often forgotten** | “I can´t because I have bad feet.” (Lucy,FG1_UK) |
|  |  | it´s a new idea yes a challenge that´s probably the best way cause it´s something you aren´t conscious about at all! I have been finding thinking that I don´t even use my toes and you really don´t´! (Louise, FG1_UK) |
|  |  | “How terribly important they are and we don´t know” (Louise, FG1_UK) |
|  |  | They don´t get looked up the same as everything else! (Anthony, FG3_UK) |
|  |  | “I… I put feet up there because I need my feet. I need my feet, you know? I need my feet, you know?” (Anthony, FG3_UK) |
|  |  | But I think most people don´t bother with feet because they´re not on show, ye know? (Anthony, FG3_UK) |
|  |  | How …How…many people have come here? But it´s feet. You know? They don´t care about feet. (Anthony, FG1_UK) |
|  |  | “People need to look after themselves…Look after their feet.” (John, FG3_UK) |
|  |  | ” Well…I tell you what I thought: Nothing! Not at all about my feet. (Jean, FG2_UK) |
|  |  | ” But I never give a thought to the fact that it could be my feet! Because when I fell I didn’t know how…I didn´t trip over anything and I was how come I´ve ended up way off to the side? That´s me coming up and being dizzy. But I didn´t think about my feet really. No I didn´t it was all…I´d lost my balance…It was either something in my ear or my head.” (Jean, FG2_UK) |
|  |  | “You´re aware of how…how…we maybe not looking at it… the bigger picture…but I am now in coming here, ye know?” (Sarah, FG3_UK) |
|  |  | “People my age so many of them are…dodgy…. In their feet…but how many listen?” (Lisa, FG2_UK) |
|  |  | “No! I didn´t think that because I don´t have other than cramps I don´t have a lot of stuff in my feet” (Mariana, FG1_PT) |
|  |  | “My toes are all crippled They don´t do the exercises well” (Vera, FG3_PT) |
|  |  | “The toes…The toes don´t grasp, that´s it.” (Maria, FG2_PT) |
|  |  | “Feet and legs have everything for someone to fall or not.” (Catarina, FG3_PT) |
|  |  | “I liked learning cause I did learn some new things ´and I just t things that I should be aware of it and I wasn´t so that was good” (Elaine, FG1_UK) |
|  |  | “It´s a new idea yes a challenge that´s probably the best way cause it´s something you aren´t conscious about at all! I have been finding thinking that I don´t even use my toes and you really don´t´!”(Louise, FG1_UK) |
|  |  | “No it´s fine…Like I said it makes your feet stronger…The back of your legs stronger. Your muscles begin to move. Maybe some muscles you never move…you haven´t used in a long time. You have a lot of muscles in ye legs, right?” (Thomas, FG2_UK) |
|  |  | ” yeah…I think that you feel the muscle whichever one it is. Well, I think to myself “oh well I must be doing some good to exercise the muscle”, otherwise you wouldn´t feel these…I don´t feel you´d feel the muscles, if you didn´t do the exercises …you are aware that those muscles are there. Well you know they are there, but you don´t feel them “(Jean, FG2_UK) |
| **Awareness about foot health, foot function and falls** |  | ” it would not rise…It was a peculiar. In the end I got it but it wasn´t every time” (Jean, FG2_UK) |
|  |  | “The most difficult ones for me were that I´ve never done exercises in barefeet… ever!” (John, FG3_UK) |
|  |  | “It´s strengthening! Strengthening your toes and ankles!” (Sarah, FG3_UK) |
|  |  | “I am not saying it´s too easy… It´s doable. It´s not something that we would do, you know? Manage to step on the heels and toes, but not yack…. I´ve done this when I was young. And ´ve done here and I thought why have I not done this before? Because it makes a difference! Even just step into my tip toes and doing my heels…”(Anthony, FG3_UK) |
|  |  | “I could see there was a little bit of a difference toward the end of the week.” (Sarah, FG3_UK) |
|  |  | “You´re aware of how…how…we maybe not looking at it… the bigger picture…but I am now in coming here, ye know?” (Sarah, FG3_UK) |
|  |  | “We should pay more because it is the same as any body part but we don´t no” (Ana, FG1_PT) |
|  |  | “Me paying more attention to the way I put my feed on the floor because lately I lose my balance very easily after I had I was a bit unwell after that fall “(Mariana, FG1_PT) |
|  |  | “When we start it´s a bit more difficult and then I think that I can´t do it, but then I keep trying keep trying and get there” (Rosa, FG2_PT) |
|  |  | “Yes. That I didn´t really think that it was as important as it is. I knew that it was important, but I didn´t know that it was as important. I´ve stopped walking in tip toes a long time ago, ever since I´ve stopped…”(Catarina, FG3_PT) |
|  |  | “No, I didn´t think about it a lot. No one had explained it to me before. That walking on your tip toes or heels could be so important for balance.” (Vera, FG3_PT) |
|  |  | “Right, if someone does gymnastics…Here, I mean exercise for their legs , so it helps the muscles and to a better quality of life. Also because the legs…If we don´t move them, they get dormant…” (Leonor, FG3_PT) |
| **Awareness about foot health, foot function and falls** | **The important role of footwear** | “I used everything under the sun…My barefeet shouldn´t is a good idea at all.” (Louise, FG1_UK) |
|  |  | “They’re quite dangerous. [slippers]” (Elaine, FG1_UK) |
|  |  | “I need to have broad part here on the top of my toes…then…” (Lucy, FG1_UK) |
|  |  | “Do you they play a part of the problem? The flip flops ? Or...because you have to watch your toes to keep them on though.” (Jean, FG2_UK) |
|  |  | “So in the house then is it better to wear this sort of thing, rather than a slipper?” (Jean, FG2_UK) |
|  |  | “Because slippers tend to stretch, don´t they?” (Jean, FG2_UK) |
|  |  | “Talking about nails…When we wear a very tight shoe or that hurts our foot. The nail is hurt, but that doesn´t mean that there is after…That it can be a problem. The nail needs to be treated. It´s different of whom really has that tendency to…To lose their nails or such.” (Amélia, FG3_PT) |
|  |  | Yes…with…a wide base of support. Right, with the problems I have on my spine.” (Leonor, FG3_PT) |

**Appendix 4**

# **Reporting checklist for qualitative studies**

Based on the SRQR guidelines.

O'Brien BC, Harris IB, Beckman TJ, Reed DA, Cook DA. Standards for reporting qualitative research: a synthesis of recommendations. Acad Med. 2014;89(9):1245-1251.

|  |  | Reporting Item |
| --- | --- | --- |
| **Title** |  |  |
|  | [#1](https://www.goodreports.org/srqr/info/#1) | Complete |
| **Abstract** |  |  |
|  | [#2](https://www.goodreports.org/srqr/info/#2) | Complete |
| **Introduction** |  |  |
| Problem formulation | [#3](https://www.goodreports.org/srqr/info/#3) | Complete |
| Purpose or research question | [#4](https://www.goodreports.org/srqr/info/#4) | Complete |
| **Methods** |  |  |
| Qualitative approach and research paradigm | [#5](https://www.goodreports.org/srqr/info/#5) | Complete |
| Researcher characteristics and reflexivity | [#6](https://www.goodreports.org/srqr/info/#6) | Not provided, as it is not disclosed in other qualitative papers in this journal. |
| Context | [#7](https://www.goodreports.org/srqr/info/#7) | Complete |
| Sampling strategy | [#8](https://www.goodreports.org/srqr/info/#8) | Complete |
| Ethical issues pertaining to human subjects | [#9](https://www.goodreports.org/srqr/info/#9) | Complete |
| Data collection methods | [#10](https://www.goodreports.org/srqr/info/#10) | Complete |
| Data collection instruments and technologies | [#11](https://www.goodreports.org/srqr/info/#11) | Complete |
| Units of study | [#12](https://www.goodreports.org/srqr/info/#12) | Complete |
| Data processing | [#13](https://www.goodreports.org/srqr/info/#13) | Complete |
| Data analysis | [#14](https://www.goodreports.org/srqr/info/#14) | Complete |
| Techniques to enhance trustworthiness | [#15](https://www.goodreports.org/srqr/info/#15) | Complete |
| **Results/findings** |  |  |
| Syntheses and interpretation | [#16](https://www.goodreports.org/srqr/info/#16) | Complete |
| Links to empirical data | [#17](https://www.goodreports.org/srqr/info/#17) | Complete |
| **Discussion** |  |  |
| Integration with prior work, implications, transferability and contribution(s) to the field | [#18](https://www.goodreports.org/srqr/info/#18) | Complete |
| Limitations | [#19](https://www.goodreports.org/srqr/info/#19) | Complete |
| **Other** |  |  |
| Conflicts of interest | [#20](https://www.goodreports.org/srqr/info/#20) | Complete |
| Funding | [#21](https://www.goodreports.org/srqr/info/#21) | Complete |

The SRQR checklist is available online at [EQUATOR Network](https://www.equator-network.org) .
